# Biomimetic Receptors for Bioanalyte Detection by Quartz Crystal Microbalances — From Molecules to Cells ^†^

**DOI:** 10.3390/s141223419

**Published:** 2014-12-05

**Authors:** Usman Latif, Jianjin Qian, Serpil Can, Franz L. Dickert

**Affiliations:** 1 Department of Analytical Chemistry, University of Vienna, Waehringer Strasse 38, Vienna A-1090, Austria; E-Mails: usmanlatif@ciit.net.pk (U.L.); Jianjin.qian@univie.ac.at (J.Q.); serpil.can@univie.ac.at (S.C.); 2 Department of Chemistry, COMSATS Institute of Information Technology, Tobe Camp, University Road, Abbottabad 22060, Pakistan

**Keywords:** estradiol, endocrine disrupting chemicals (EDCs), quartz crystal microbalance (QCM), molecular imprinted polymers (MIPs), *Escherichia coli* (*E. coli*), *Bacillus subtilis* (*B. subtilis*)

## Abstract

A universal label-free detection of bioanalytes can be performed with biomimetic quartz crystal microbalance (QCM) coatings prepared by imprinting strategies. Bulk imprinting was used to detect the endocrine disrupting chemicals (EDCs) known as estradiols. The estrogen 17β-estradiol is one of the most potent EDCs, even at very low concentrations. A highly sensitive, selective and robust QCM sensor was fabricated for real time monitoring of 17β-estradiol in water samples by using molecular imprinted polyurethane. Optimization of porogen (pyrene) and cross-linker (phloroglucinol) levels leads to improved sensitivity, selectivity and response time of the estradiol sensor. Surface imprinting of polyurethane as sensor coating also allowed us to generate interaction sites for the selective recognition of bacteria, even in a very complex mixture of interfering compounds, while they were growing from their spores in nutrient solution. A double molecular imprinting approach was followed to transfer the geometrical features of natural bacteria onto the synthetic polymer to generate biomimetic bacteria. The use of biomimetic bacteria as template makes it possible to prepare multiple sensor coatings with similar sensitivity and selectivity. Thus, cell typing, e.g., differentiation of bacteria strains, bacteria growth profile and extent of their nutrition, can be monitored by biomimetic mass sensors. Obviously, this leads to controlled cell growth in bioreactors.

## 1. Introduction

Living systems are the source of inspiration for scientists seeking to design sensors, separation devices, and drug delivery systems. In living systems, the proper functionality of certain biological operations is dependent on specific recognition events at a molecular level and it is a key necessity for the integrity of living cells. Imitation of natural methods, mechanisms, and processes occurring in living things is the best way to extract the natural selectivity that makes living things the perfect machines that no one is capable of developing [1]. Scientists are trying very hard to imitate the recognition properties of biological systems on artificial platforms to selectively detect or separate analytes of interest. Thus, a large number of attempts have been made by researchers in developing man-made materials with biomimetic properties. These biomimetic architectures possess the recognition properties of biological systems and are able to detect viruses [2], bacteria by biomimetic chemical reduction [3], trace level biological signaling molecules from living cells [4], superoxide anions for living cell monitoring [5], of classify honeys and detect their adulteration [6]. This biomimetic approach also makes it possible to develop sensor devices for the detection of carcinoma antigens [7], copper ions by biomimetically synthesized quantum dots [8], and circulating tumor cells (CTCs) by biomimetic surfaces functionalized with epithelial-cell adhesion molecule (EpCAM) [9,10].

Biomimetic colorimetric sensors were also developed using genetically engineered viruses to introduce selectivity for the detection of pathogens and harmful toxicants to protect human health and national security [11]. Enhanced selectivity of biomimetic architectured immunoassays is also attributed to virus-tethered magnetic gold microspheres [12]. Integration of modern nanoelectronic technology with the potent molecular machines of living organisms offers a pathway towards the development of bioelectronic (biomimetic) noses that can be directly compared with biological olfactory systems [13] or biomimetic tongues based on metal-organic frameworks [14]. Biomimetic membranes owing their selectivity to the biological membranes that define boundaries and organelles in living cells on which the integrity of cells depends were developed for sensors, separation devices, drug delivery systems, and nanocontainers [15–17]. Thus, the imitation of natural mechanisms ensures highly selective, sensitive, robust, and low cost biomimetic materials for a large number of applications.

The recognition properties of natural receptors can easily be reproduced in man-made materials by following a straightforward, very powerful, and universally adaptive technique such as molecular imprinting to generate artificial receptors. Biomimetic polymers based on the molecular imprinting (MIP) approach exhibit high selectivity and affinity towards target molecules [18–21]. These biomimetic materials with required recognition properties can by synthesized by following either a bulk imprinting or surface imprinting approach. In MIP synthesis via the bulk imprinting route, a functional monomer is copolymerized with a cross linker in the presence of a template to form a rigid polymer network. The combination between functional monomer and template in synthesizing a biomimetic polymer is similar to the bonds of natural biological receptors. Afterwards, the template is removed either by washing or evaporation, which leaves behind recognition sites responsible for the selective and reversible inclusion of the template compounds due to their steric adaptation. Surface imprinting is a four step process: deposition of a thin layer of template on a glass slide (first step), coating a pre-polymer on a transducer surface (second step), stamp fixation on the pre-polymer until complete polymerization in which the polymer matrix arranges itself around the template (third step) whereas in the last step the stamp is removed which leaves behind recognition sites for reversible inclusion of a templated analyte. The choice of the bulk or surface imprinting approach in generating artificial receptors depends totally on the target analyte. The bulk imprinting approach will generate interaction cavities in a polymer matrix which are highly suitable for detecting small molecules, whereas surface imprinting is a promising technique for detecting larger analytes such as bacteria or viruses. The removal of the template during polymerization after bulk imprinting will produce diffusion pathways for the reversible inclusion of small molecules, whereas the large dimensions of microorganisms, ranging up to few micrometers, would hinder their diffusion into the cavities produced by bulk imprinting. MIPs have been applied successfully as biomimetic receptors in various fields such as solid phase extraction [22], development of sensors for various targets based on different transduction principles [23–30], drug delivery vehicles [31,32], induced crystallization [33] and in other different bioapplications [34]. The most prominent feature of these MIPs is their robustness which helps in creating sensor devices with regeneration and reusability properties. The quartz crystal microbalance (QCM) is a high resolution mass sensitive transducer which can measure a change in the mass of a target analyte by monitoring a variation in the oscillation frequency of the quartz crystal in real time [35–38]. The combination of the QCM technique with MIPs provides label-free, selective, sensitive, low cost, simple and stable detection systems. This combination has already been used in determination of polycyclic aromatic hydrocarbons, and the detection of viruses [39], blood cells [40], enzymes, and proteins [41].

Estradiol is one of the known endocrine disrupting chemicals (EDCs) which cause adverse effects on the endocrine system of humans and wild animals [42]. A large number of substances such as estrogens, progestrogens, synthetic estrogens, different organic pollutants, pesticides, and surfactants are known as EDCs [43]. Steroid estrogens like estrone (E1), 17β-estradiol (E2), estriol (E3), and 17β-ethinylestradiol (EE2) are the most familiar EDCs [44]. Naturally occurring sulfate and glucuronide conjugates of estrogens such as E2, and its metabolites E3 and E1 are largely excreted from the human body via the urine [45]. These estrogens are frequently found either in sewage effluents or in environmental waters and pose a great threat to aquatic life and public health [46,47]. Major concerns about the presence of estradiol, even in a low concentration, are due to the fact it causes growth abnormalities, disturbs reproductive system functions, and has breast tumor implications [48–50]. Thus, it is very important for public and environment health to create a device which can detect estradiol online in organisms, foods or environment.

In recent years, major concerns have emerged around the world due to pathogenic bacteria which represent a great threat to public health [51,52]. Continual outbreaks of pathogenic bacteria have caused lots of panic and illnesses to millions of peoples and are even considered responsible for thousands of deaths [53–56]. The worst pathogenic *E. coli* strains can cause bloody diarrhea, hemorrhagic colitis, and hemolytic uremic syndrome and sometimes are responsible for kidney failure leading to death [57–60]. Pathogenic strains of *E. coli* are a leading cause of deaths in children of age less than 5 years which is estimated to cause some 1.3 million deaths worldwide [61]. The Gram-positive rod shaped species of Bacillus genus such as *Bacillus anthracis, Bacillus subtilis, Bacillus thuringiensis*, and *Bacillus cereus* produce endospores in response to nutrient deprivation [62,63]. These endospores can withstand radiation, heat and other harsh conditions for a long time and produce their respective bacteria cells under appropriate conditions [64,65]. The endospores of pathogenic species can cause different diseases such as tetanus, anthrax, and botulism if acquired by a host via the skin, inhalation or by ingestion [66,67]. These pathogenic species are a great threat to human life. Recent studies have shown that Bacillus species are genetically similar to each other, so, we used *Bacillus subtilis* as a model species to develop a sensor for monitoring the growth of harmful bacteria from their endospores.

In this study, we have prepared a QCM sensor for estradiol by using an E2 imprinted polyurethane as sensitive layer to recognize the template (E2) selectively, even in the presence of its structural analogues. Optimization of cross linker and incorporation of porogens in the MIP polyurethane not only helps increase the sensitivity and selectivity towards the targeted analyte, but also decreases its response time by improving its diffusion pathways. A surface imprinting approach was also followed to develop artificial bacteria cells by which an imprinted polyurethane was prepared for selective detection and differentiation of bacterial cell strains. Bacterial endospores are dormant structures produced by stressed bacteria cells. These endospores are capable of transforming into highly dangerous bacteria with proper nutrition. The surface imprinting strategy was also utilized to develop a sensor for investigating *Bacillus subtilis* bacteria growth from the respective spores.

## 2. Experimental Section

All reagents and solvents were purchased in a highest available purity and used without further processing. The monomers 4,4′-methylenediphenyl diisocyanate (MDI), poly(4-vinylphenol) (PVP), 4,4′-dihydroxy-2,2-diphenylpropane (bisphenol A, BPA) and solvent (tetrahydrofuran, THF) were purchased from Merck Chemicals and Life Science GesmbH (Vienna, Austria). The cross linker 1,3,5-trihydroxybenzene (phloroglucinol, PG), and pyridine catalyst were purchased from Fluka (Vienna, Austria). The artificial bacterial cells were prepared by using polydimethylsiloxane (PDMS). The required silicone elastomer kit (Sylgard 184 silicone kit) was obtained from Dow Corning (Wiesbaden, Germany). Bacteria *E. coli* strain W (ATCC 9637), strain B (EC 11303) and *Bacillus subtilis* (ATCC 6633) were purchased from Sigma-Aldrich Handels Gmbh (Vienna, Austria). AT-cut quartz crystals with 15.5 mm diameter and 168 μm thickness were purchased from Zhejiang Quartz Crystal Corporation (Taizhou, China). Electrodes were screen printed on the quartz crystal microbalance (QCM) using GGP 2093-12% brilliant gold paste (Heraeus, Hanau, Germany).

### 2.1. Estradiol Imprinting

A biomimetic receptor was generated by templating a polyurethane layer with estradiol. The polyurethane solution was prepared from stock-solutions of 100 mg of each ingredient in 1 mL tetrahydrofuran by mixing 20 μL of 4,4′-methylenediphenyl diisocyanate (MDI), 30 μL of poly(4-vinylphenol) (PVP), 30 μL of phloroglucinol (PG) as linker and 100 μL of tetrahydrofuran (THF) as solvent. A volume of 3 μL of E2 was added as template and 5 μL pyrene as porogen. A volume of 15 μL of pyridine was added to start polymerization, which occurred at 70 °C for 15 min until the gel point was reached. Polyurethane formation or reaction completion can be confirmed by monitoring the isocyanate band that gives a prominent peak at 2263 cm^−1^ in the IR which diminishes quickly near the gel point. The estradiol imprinted polyurethane was further diluted 1:8 with THF for spin coating on a transducer (QCM) surface and left for 48 h for complete polymerization. E2 was washed out from imprinted polyurethane layer (coated on QCM) by dipping the QCM in 30 mL of water and stirring for 30 min. Subsequently, a volume of 1 mL of acetonitrile was added to the resulting aqueous solution of E2, after concentration of the aqueous solution. The β-estradiol in this solution was determined by a Perkin-Elmer LS50B luminescence spectrometer and recorded at an excitation and emission wavelength at 283 nm and 304 nm, respectively. The β-estradiol was analyzed via a linear calibration curve in the concentration range of 0.1 to 5.0 μg/mL in acetonitrile. Three washing steps were performed and only 6% estradiol was detected after the second washing step, whereas the third extraction ensures 0% of estradiol content.

### 2.2. Artificial E. coli Bacteria

The polyurethane was synthesized by reacting 4,4′-dihydroxy-2,2-diphenylpropane (BPA, bisphenol A), and 4,4′-methylenediphenyl diisocyanate (MDI) as monomers and phloroglucinol (PG) as crosslinker in the following ratio: 47.8 mg, 40.2 mg, and 12 mg, respectively. A volume of 100 μL of THF was used as solvent and 15 μL pyridine as a catalyst. The mixture of monomers and crosslinker was heated at 70 °C until the gel point was reached. This pre-polymerized mixture was diluted with THF up to 1000 μL. A volume of 15 μL of this diluted pre-polymerized mixture was spin-coated on 8 × 8 mm glass slides (roughness approximately 2 nm; Schott, Mainz, Germany) at a spinning rate of 2500 rpm. This spin-coated pre-polymer thin layer was surface imprinted by using *Escherichia coli* (*E. coli*) bacteria. For this purpose, a stamp was prepared by depositing a thin film of natural bacterial cells on 8 × 8 mm glass slides. The stamp of natural cells (template) was pressed onto the pre-polymer with a clamp and left for 48 h in air to ensure complete polymerization. Stamp and template were removed from the polyurethane layer by dipping it in ultrasonic bath until they separated. This process generates cavities on the surface of the polyurethane thin film complementary to *E. coli* bacteria.

An artificial bacteria stamp was generated by casting polydimethylsiloxane (PDMS) in the cavities of the imprinted polyurethane. A Sylgard elastomer kit (Dow Corning Wiesbaden, Germany) was used to produce the silicone polymer and the required rigidity was achieved by applying the base material and a hardener in a ratio 10:1. The *E. coli* imprinted polyurethane layer was covered by this silicon polymer and left for 48 h at room temperature to ensure complete polymerization. In this process, the geometrical features of *E. coli* bacteria are transferred to the silicon material. This silicon polymer was then peeled off from imprinted polyurethane layer and used as an artificial *E. coli* bacteria (*E. coli* replica) stamp.

Finally, 15 μL of diluted pre-polymer (polyurethane) was spin-coated on the quartz electrode at a spinning rate of 2500 rpm. Then, the artificial *E. coli* bacteria stamp was pressed on the prepolymer-coated QCM with a clamp and left for 48 h at room temperature to ensure complete polymerization. Finally, the *E. coli* replica stamp was removed from the polyurethane by immersing it in an ultrasonic bath until it separated.

### 2.3. Bacteria Growth Sensor

A *Bacillus subtilis* (*B. subtilis*) growth sensor was prepared by surface imprinting the polyurethane layer with *B. subtilis*. For this purpose, a bacterial stamp was formed by assembling *B. subtilis* on 8 × 8 mm glass slides. Polyurethane was prepared at 70 °C from 40.2% MDI, and 47.8% PVP as monomers and 12% PG as cross linker until the gel point is reached while 100 μL of THF was used as solvent and 15 μL pyridine as a catalyst. This prepolymer is further diluted with THF up to 1000 μL. A volume of 15 μL of diluted prepolymer was spin-coated on the quartz electrode at a spinning rate of 2500 rpm. Then, the bacteria stamp is pressed on this pre-polymer polyurethane and allowed to stand overnight to align the polymer around the template, which will generate the cavities for reversible inclusion of analyte, after removing the bacteria stamp with water in an ultrasonic bath.

### 2.4. Measurements

The mass sensitive measurements were performed by a dual electrode (a measuring and a reference electrode) quartz crystal microbalance (QCM) of frequency 10 MHz having 15.5 mm diameter and thickness of 0.168 mm. The electrodes were coated on a single AT-cut quartz disc by screen printing with gold paste (Heraeus) and subsequently, heated at 400 °C for 3 h. These mass sensitive QCM devices allow mass detection of analytes down to nanogram levels. The dual electrode quartz sensor is placed into a microfluidic chamber in order to reduce the analyte diffusion path which in turn reduces the sensor response time. In this setup, a quartz plate is sandwiched between two polydimethylsiloxane (PDMS) layers. The upper layer has an inlet and outlet for analyte exposure to the electrodes, whereas, the bottom layer consists of a chamber filled with air which allows proper oscillation of the quartz sensor. The capacity of this measurement cell can accommodate 140 μL or less analyte volume. The serial resonance of quartz sensor was monitored by a customized amplifier. All measurements were repeated three times. The sensor responses of the newly fabricated imprinted QCMs were all within 10% of the frequency responses. The noise in the sensor response was within 1 Hz to 5 Hz which depends on thickness and roughness of the sensor coating. The dominant contribution of frequency fluctuations are due to temperature alterations. These fluctuations were compensated by differential measurements. Light microscopy and contact mode atomic force microscopy (AFM) was used for surface characterization. A Perkin-Elmer LS50B luminescence spectrometer was employed for estradiol detection.

## 3. Results and Discussion

### 3.1. Estradiol Sensor

The use of polyurethanes was extraordinarily successful for this task. This polymer shows hydrophobic interactions due to presence of aromatic rings in its network. The phenolic reactants were added in excess which creates additional hydrophilic interaction centers. Quartz crystal microbalances (QCMs, QMBs) with a fundamental frequency of 10 MHz were used since they are easy to handle and can detect mass down to one nanogram. The polyurethanes were prepared from MDI, BPA as monomer and PG as cross-linker. The estrogen of interest was added as template during the polymer synthesis. The mixture was polymerized to a gel point suitable for coating QCM followed by curing of the material.

**Figure 1.**
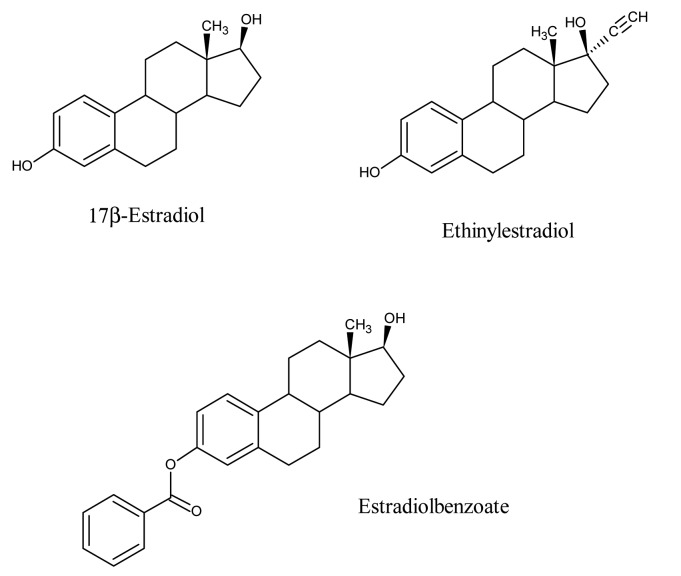
Chemical structures of estradiol (E2), ethinylestradiol (EE2) and estradiol benzoate (E2B).

The host-guest chemistry strategy was checked by fluorescence spectroscopy. For this purpose, the template (E2) was extracted from the sensor layer in aqueous solution. The estradiol in aqueous solution was determined by fluorescence spectroscopy. These preliminary investigations reveal that half of the template molecules are not washed out, which is attributed to a lack of proper diffusion pathways. This problem was addressed later on by adding porogens during MIP synthesis to introduce adequate diffusion pathways for washing and reversible inclusion of template. However, measurements with QCMs were performed based on these preliminary results to test the amount of E2 which was incorporated by sensor coating. The frequency response of one Hertz corresponds to one nanogram of layer incorporated with estradiol according to the Sauerbrey equation. The imprinting with E2 leads to an effect of 33 Hz while exposed to an analyte concentration of 2.5 mg/L in water. Only a part of the available cavities are occupied at this concentration. This finding was confirmed by a nearly linear concentration dependence of the sensor response, so obviously this sensor showed no saturation. The imprinting success was confirmed by selectivity studies. Thus, estradiol structural analogues such as estradiol benzoate and ethinylestradiol are exposed to a sensor imprinted with E2. The chemical structures of some estradiols which differs slightly from each other are shown in Figure 1.

Appreciable differences in sensor responses for 17β-estradiol (33 Hz), estradiol benzoate (13 Hz) and ethinylestradiol (4 Hz), were observed. The sensor responses differed significantly despite the fact they have a similar scaffold and only differ in the substituents. This is due not only to the geometry of the cavity but also the interactions with the functional groups. Approximately, 55% of the E2 was washed out from the E2-imprinted polymer matrix as measured by luminescence spectrometry. Additionally, a number of available cavities were unoccupied by the analyte in equilibrium with the aqueous solution of E2. These effects are actually responsible for a lower sensor response of 33 Hz to 2.5 mg/L estradiol in water.

**Figure 2.**
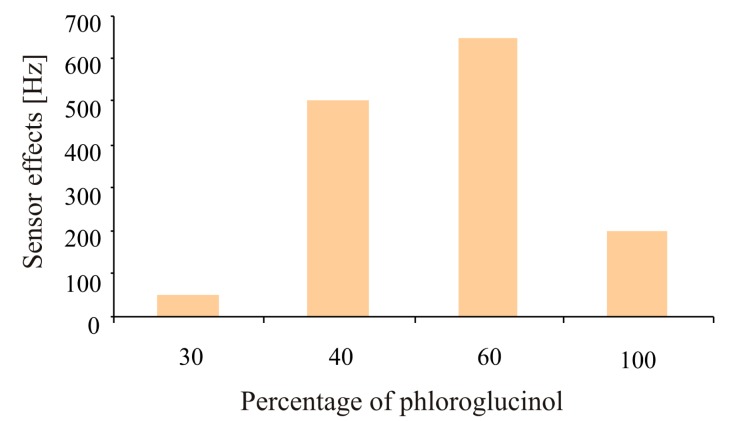
Sensor effects against E2 as a function of phloroglucinol content in polyurethane.

Further strategies were developed to improve the adsorption-desorption behavior of the imprinted layer (cavities) to achieve enhanced sensitivity. This can be done by optimization in two respects. On the one hand a systematic variation of the phloroglucinol cross linker was performed. An optimized binding of the analyte by the polymer was achieved without blocking the analyte diffusion pathways. The amount of added phloroglucinol was varied in a percentage between 30%–100% (with respect to the polymer building blocks). A cross linker of 60% enhanced the sensor effect by approximately a factor of 10 in respect to the minimum response as shown in Figure 2. A content of 100% phloroglucinol guaranteed the highest stability of the molecular cavities generated by imprinting.

On the other hand the addition of porogens improved the sensitivity by ensuring analyte diffusion in the polymer. The improvement in diffusion pathways not only favors the binding of analytes with recognition sites, but also makes it easy to recover the sensor response. Thus, there is a substantial improvement in the reversible inclusion behavior of analytes.

**Figure 3.**
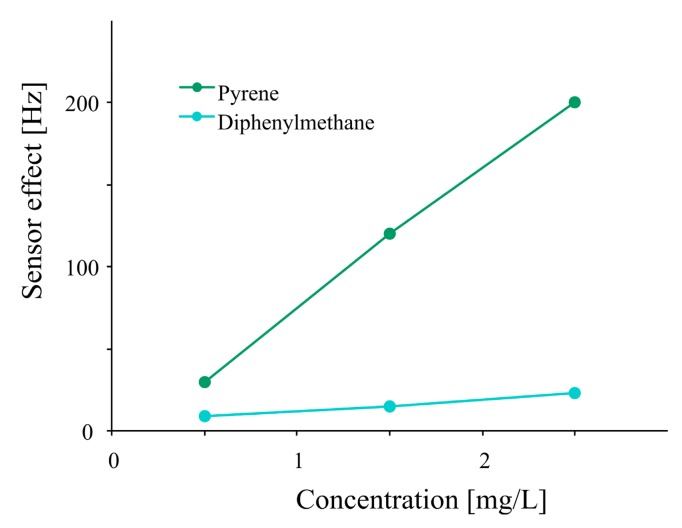
Sensor effects as a function of β-estradiol concentrations after incorporating porogens (pyrene, diphenylmethane) in molecular imprinted polyurethane during synthesis.

Two molecules without functionality which are not covalently adhered by the polymer were chosen. Diphenylmethane is suitable for this purpose, due to its geometrical similarity to the isocyanate and was effectively included in the polymer. Pyrene with an extended π-system was also excellently incorporated in polyurethane. A higher sensor response was obtained while using pyrene as porogen in comparison to diphenylmethane, as shown in Figure 3. Optimization of imprinted polyurethane adsorption-desorption behavior by changing cross-linker percentage (Figure 2) and addition of pyrene as porogen (Figure 3) resulted in a sensor response of about 500 Hz to 2.5 mg/L of E2 which is an improvement by factor of 20 in respect to former measurements.

**Figure 4.**
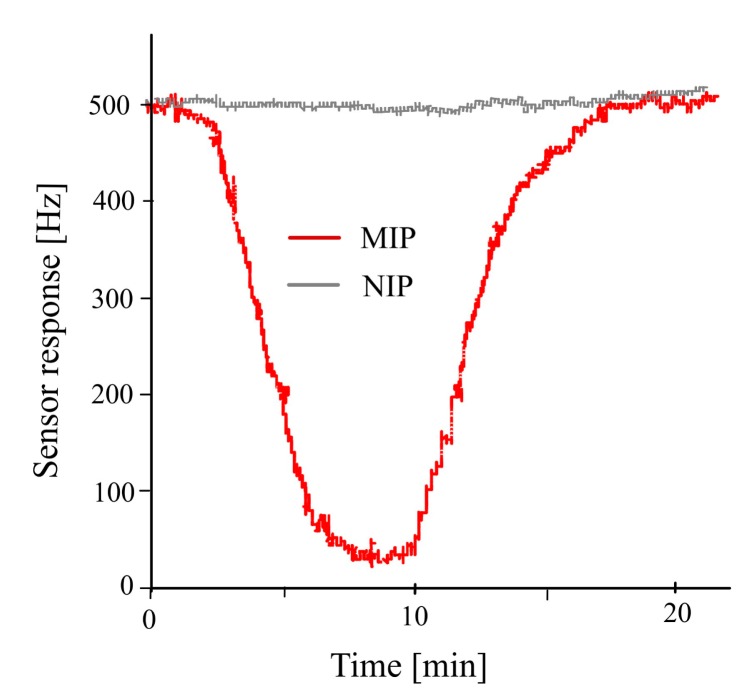
Sensor responses of estradiol imprinted polyurethane against its templated analyte (estradiol). Non-imprinted polyurethane layer served as reference for differential measurements.

β-Estradiol imprinted polyurethane sensor was exposed to its templated analyte E2 after adjusting all necessary parameters such as amount of cross-linker as well as type and concentration of porogen as shown in Figure 4. Water was used for flushing out the included analyte from the sensor coating which makes the frequency of the sensor return back to baseline. The adequate response time of the sensor is attributed to the addition of porogen which generates the required diffusion pathways for reversible inclusion of analyte molecules. Non-imprinted polyurethane layer having a similar ratio of cross-linker and porogen was used as reference for differential measurements. A negligible frequency response of the reference electrode against different analytes in comparison to the imprinted polyurethane clearly depicts that how successful the imprinting is in order to detect its templated analyte.

**Figure 5.**
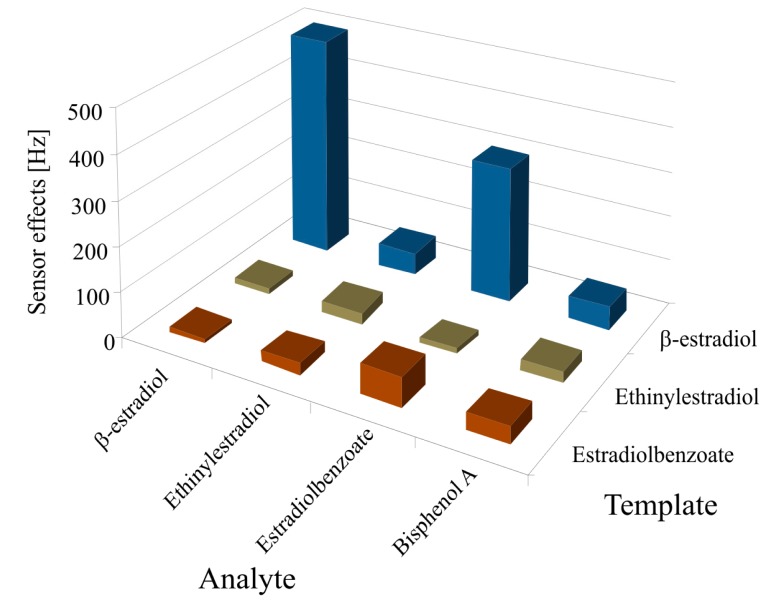
Selectivity pattern between β-estradiol (E2), and its structural analogues such as estradiolbenzoate (E2B) and ethinylestradiol (EE2). Sensor responses were also obtained by exposing to environmental estrogen, bisphenol A, which also affects the hormone system.

Furthermore, the efficiency of imprinted polyurethane was also investigated by measuring the selectivity pattern between E2, and its structural analogues such as E2B and EE2. The polyurethane layers were imprinted with E2, E2B and EE2 and exposed to these templated analytes as shown in Figure 5. They show very low sensitivity against all sensors, especially β-estradiol imprinted sensor. The cross sensitivity profile suggested that the most significant response was obtained for those analytes which are identical to the template.

A pronounced sensor response on E2 imprinted sensor was observed for its templated analyte E2, whereas other prominent sensor responses were shown by the E2 structural analogue E2B because the only difference in their structures is a benzoate group. The sensor effect of other structural analogue of estradiol, EE2, is almost negligible. A sensor response was also obtained for BPA, an environmental estrogen, because this phenol is known to affect the hormone system, influencing the metabolism of endogenous steroids, drugs and other xenobiotics [68]. Aqueous solutions of estrogens were measured which clearly indicated that E2 concentrations in sewage water can easily be identified selectively, even in the presence of its structural analogues and BPA.

It is evident from all these studies that imprinted polyurethane is a suitable polymer to create selective receptors for estradiols. In comparison to polyurethane, the recognition cavities generated in polyacrylates and polystyrenes by imprinting show low sensitivity and unfortunately more non-selective adsorption.

### 3.2. Bacteria Sensor

The lithographic stamping method has also been used for the detection of bacterial cells. Master stamps which can be used for the stamping of polymers for generating sensors were prepared using artificial bacteria cells instead of consuming natural bacteria cells. There are a certain number of pathogenic bacteria which are very harmful and could be used at any time as possible bio-weapons for terrorist attacks. The tendency of bioanalytes to adapt to the actual environment and their mutation leads to a growing number of possible threats. Thus, the use of such a type of pathogenic bacteria as template can be minimized during biomimetic sensitive layer synthesis by replacing them with artificial ones. The use of dangerous bacteria cannot be avoided completely because these cells are needed only once to make their replicas. The sensors based on piezoelectric transducers along with recognition layers, made with artificial bacteria, present a suitable solution to avoid these threats. The main advantages of these sensors are selectivity, sensitivity, fast response times (on the time scale of seconds) and easy handling.

An improvement in reproducibility can also be achieved by using artificial cells for the generation of these sensitive layers. Bacteria-chips were produced and their functionality was tested and optimized by combining QCMs with a sensitive layer. Polyurethanes are optimized polymers which can be used for soft-lithographic imprinting.

**Figure 6.**
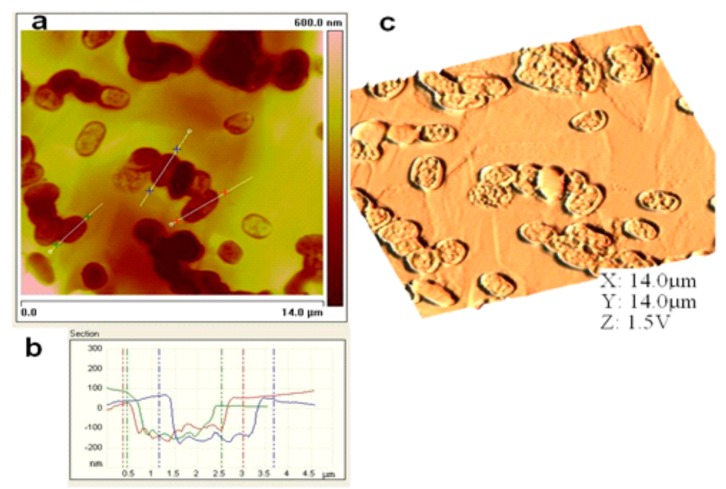
Polyurethane sensitive layer having cavities for reversible inclusion of *E. coli*, imprinted with a synthetic bacteria stamp (replica of *E. coli* W) made of silicon (**a**) 2-dimensional presentation of the surface with AFM—contact mode; (**b**) Section analysis: depth profile of the indicated cavities in figure (a); (**c**) 3-dimensional presentation of the polymer section from figure (a), graphical processing of the results with the program WSxM.

Atomic force microscopy (AFM) is a useful tool to characterize the sensor surface. The generation of recognition cavities by templating the surface ensures the reversible incorporation of analytes which is a prerequisite parameter for mass-sensitive detection. The inclusion of bioanalytes in these cavities results in producing sensor responses when exposed to sample solutions. A bacteria sensor for *Escherichia coli* strain W was designed by using artificial cells of *E. coli* W. First, the polyurethane layer is templated with natural cells and then a silicon stamp of artificial strain W cells (replicas of *E. coli* strain W) was prepared by covering these imprinted sites with a silicon layer. This silicon layer was separated from the structured layer after complete polymerization and later used as an artificial bacterial stamp for templating. Figure 6a,c represent the 2D and 3D AFM images of the cavities in the polyurethane layer generated by these artificial bacterial cell stamps and the image in Figure 6b is a measurement of the depth profile of these cavities.

**Figure 7.**
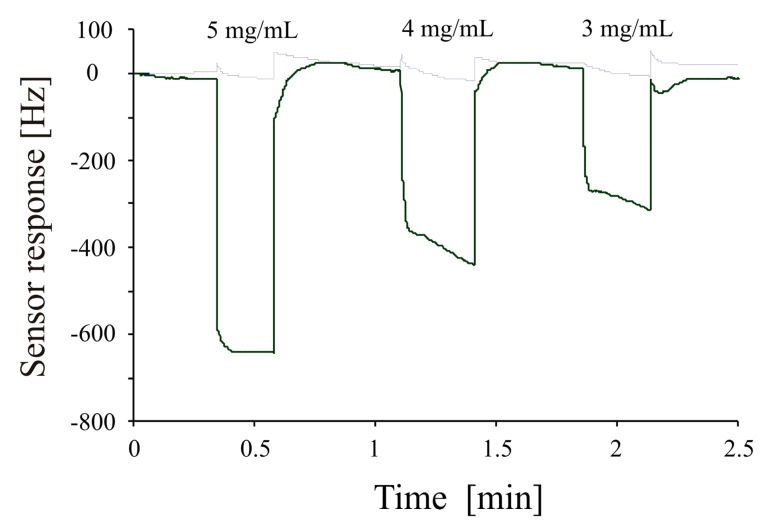
Concentration dependent frequency response of polyurethane layer imprinted with *E. coli* strain W replica (1 mg of bacteria approximately corresponds to 5 × 10^8^ cells).

This surface characterization reveals that the geometrical features of *E. coli* W are transferred to the sensitive layer via artificial cell stamping instead of using natural cells. The imprinting of polyurethane with these plastic cells generates a sensor surface with similar properties as those prepared from native cells. The plastic cells, however, show a higher robustness than native cells. Furthermore, they can be used for producing a large number of bacteria sensors by utilizing a single stamp. The sensitivity of the bacterial sensor generated by using an artificial bacteria strain stamp for structuring the sensor surface, was investigated by mass sensitive measurements on a dual electrode 10 MHz QCM. Differential measurements were carried out to avoid non-selective responses by covering one channel of the dual electrode QCM with imprinted polyurethane layer (templated with artificial *E. coli* W) and using the second channel as reference. Figure 7 shows the concentration dependent frequency response of the *E. coli* W templated sensor when exposed to 3 mg/mL to 5 mg/mL of *E. coli* W samples. This picture clearly shows successful imprinting of the polyurethane layer as evident by the sensitivity pattern of the sensor. The frequency of the bacterial mass sensor drops immediately when exposed to different concentrations (fast response time) and quickly returns back to baseline when flushed with water (reusability).

**Figure 8.**
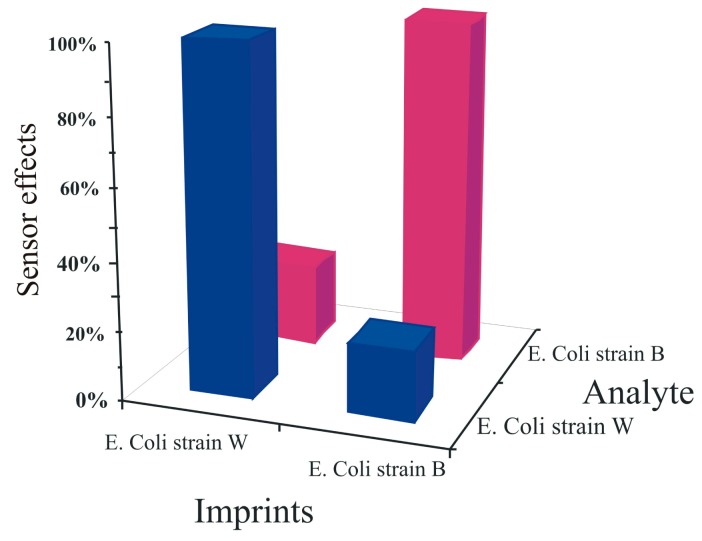
Cross sensitivity of a *E. coli* bacteria sensor, 10 MHz QCM coated with polyurethane and imprinted with synthetic *E. coli* W and B stamps sensitive to strain W, and strain B, respectively. *E. coli* B and W having a concentration of 5 mg/mL in water were measured while exposing to different sensors at room temperature.

The imprinting pattern produced by the artificial *E. coli* stamp was assessed by measuring the cross sensitivities of these bacterial sensors against different strains of *E. coli*. For this purpose, two bacterial sensors were prepared by surface imprinting: one sensor surface was templated with artificial *E. coli* W stamp whereas, the second with imprinted with the artificial *E. coli* B stamp. These sensor devices were exposed to equal concentrations (5 mg·L^−1^) of both *E. coli* B and W under similar conditions, as shown in Figure 8.

The results show that the *E. coli* strain B templated sensor responds strongly to its respective strain and same is the case with the strain W templated sensor whereas these sensors present very minimum responses to the opposite strains. These findings show the selectivity of the sensor responses and clearly represent that the selectivity patterns have been transferred successfully to the sensitive layer even by using artificial cells for generating recognition cavities on the sensor surface instead of using natural *E. coli* cells.

### 3.3. Bacteria Growth Sensor

**Figure 9.**
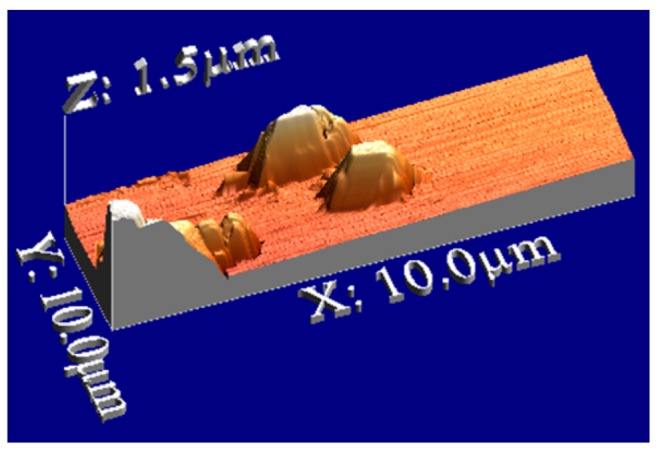
Surface characterization of *Bacillus subtilis* spores by atomic force microscopy in contact mode.

The potential hazards associated with pathogenic bacteria and their use for terrorist and military purposes, challenges the development of sensors for online monitoring. Pathogenic bacteria such as *Bacillus anthracis* can cause anthrax, a possible bio-threat, which can be avoided if properly monitored. A Bacillus bacterium has the capacity to produce endospores in response to nutrient deprivation, thus it is equally important to measure spores and their subsequent transformation into the respective bacteria. These endospores can withstand harsh conditions and can be transformed into highly dangerous bacteria with proper nutrition. It is not wise to use *Bacillus anthracis* as a template because of the potential hazards associated with this species, so the spores and bacteria of *Bacillus subtilis* were used as a model organism to develop a sensor device for *Bacillus anthracis* (anthrax). The budding of spores can be monitored by classical microscopy or an atomic force microscope (AFM). An AFM image of *Bacillus subtilis* (*B. subtilis*) spores is shown in Figure 9.

**Figure 10.**
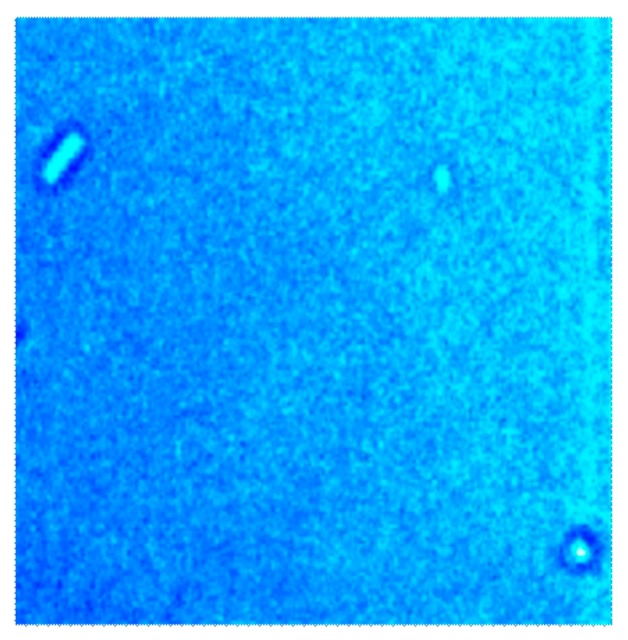
The transformation of *Bacillus subtilis* spores into the respective bacteria analyzed by light microscopy.

The spores of *Bacillus subtilis* (*B. subtilis*) will grow into the respective bacteria in the presence of nutrient solution. The spores of *B. subtilis* were added to a nutrient solution (PBS with 2% ammonium sulfate and 10% glucose) at temperature 42 °C and pH 7.2. The transformation of these spores into the respective bacteria, after an incubation period of 7 h, can be seen by a light microscope as shown in Figure 10.

**Figure 11.**
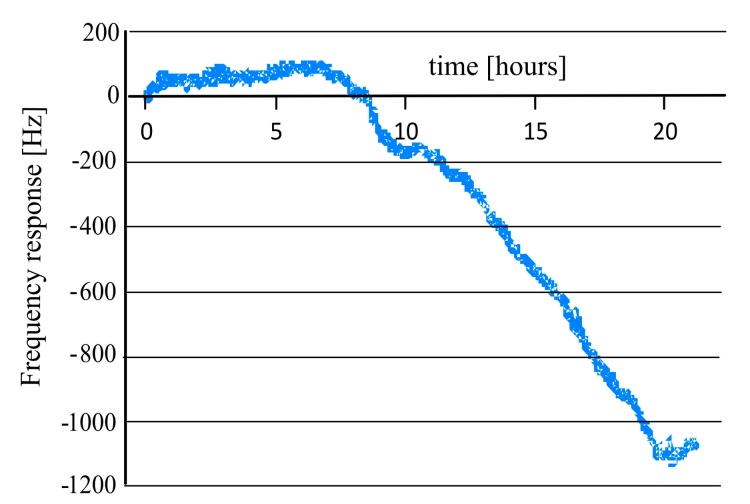
Measuring the growth of *Bacillus subtilis* from its spores by *B. subtilis* imprinted polyurethane, in nutrition solution of 2% ammonium sulfate and 10% glucose at temperature 42 °C and pH 7.2.

The growth of *Bacillus subtilis* (*B. subtilis*) from its spores was monitored by a mass sensitive sensor. A dual electrode QCM transducer used as a mass sensitive sensor for *B. subtilis* was manufactured by coating with a *B. subtilis* imprinted polyurethane layer. In this case, the surface imprinting process was carried out to transfer the features of the bacteria to the polymer surface which produces *B. subtilis* recognition cavities on the sensor surface. A non-imprinted polyurethane layer was used as reference, in order to avoid any non-selective interactions in the liquid phase. The spores of *B. subtilis* were added in a nutrient solution (PBS with 2% ammonium sulfate and 10% glucose) at a temperature of 42 °C and pH 7.2. The growth of *B. subtilis* from its respective spores was monitored as a function of time by exposing the QCM to spores (in nutrient solution). As the sensitive layer of the QCM is templated with *B. subtilis*, it has the ability to selectively recognize its templated analyte. The inclusion of the templated analyte (*B. subtilis*) in the recognition cavities of the polyurethane layer will decrease the frequency of the mass sensitive sensor according to the Sauerbrey principle. Thus, in the beginning, the frequency of the QCM remains almost constant for up to 7 h because the nutrient solution contains only spores, as shown in Figure 11.

After that, the frequency of the QCM goes down with the growth of bacteria from its respective spores due to inclusion of *B. subtilis* bacteria in the imprinted cavities. This measurement shows the sensitive and selective behavior of imprinted polyurethane which can recognize *B. subtilis* bacteria, even in a very complex mixture of various interfering compounds.

## 4. Conclusions

This study reveals that molecular imprinting is a universal technique to imitate natural selectivity in man-made materials. The geometrical features of the targeted analytes were successfully transferred to synthetic polymers in a process of analyte (template) immersion and subsequent removal from the polymeric network. This process leads to biomimetic sensor layers with selectivity comparable to that of natural receptors. Molecular imprinting was not only successful for generating cavities to recognize molecules such as estradiol, but also for bacteria cells. Porogens and cross linkers are equally important to design a sensor coating with high sensitivity, selectivity, adequate response time, and regeneration properties. β-Estradiol, a potent endocrine disrupting chemical, can be easily detected by imprinted polyurethane even in the presence of some of its structural analogues. The double imprinting approach is also very successful and interesting as it leads to the formation of artificial bacteria cells (replicas of bacteria cells). The artificial cells used for the generation of imprinted polyurethanes inherit the selectivity of natural cells by which these sensor surfaces are even able to differentiate between *E. coli* strains W & B. The development of biomimetic sensor devices for detection of bacterial cell growth from endospores in the presence of complex mixtures has implications for health, industry and the environment. This study demonstrates the versatility of molecular imprinting technique in designing artificial receptors not only for small molecules but also for larger analytes (bacteria). It has shown that bulk imprinting can be used for molecules whereas surface imprinting (patterning) is preferred for bioparticles.
